# Supplementary material for: Nuclear and cytosolic J-domain proteins provide synergistic control of Hsf1 at distinct phases of the heat shock response
Source: bioRxiv. 2025 Jul 8:2025.04.14.648540. Preprint. [Version 2] doi: 10.1101/2025.04.14.648540 (PMC12265559; doi:10.1101/2025.04.14.648540)
Supplement: Supplement 1 — Figure 1 – Figure Supplement 1 Attenuation of the Hsf1-mediated heat shock response is defective in S. cerevisiae apj1Δ cells. (A/B) S. cerevisiae wt and apj1Δ cells harboring an Hsf1-controlled unstable GFP reporter were grown at 30°C till logarithmic growth phase and shifted to 38°C. After 10 min Cycloheximide (CHX) was added to inhibit protein synthesis (B). Total cell extracts were prepared and levels of the heat shock protein Btn2 were determined by western blot analysis at the indicated timepoints after heat shock. Zwf1 levels were determined as loading control. (C/D) Levels of translated mRNAs of Hsf1-target genes were determined prior and post heat shock (30°C-38°C) by ribosome profiling in apj1Δ, sis1-4xcga and apj1Δ sis1-4xcga cells and were normalized to respective mRNA levels determined in wild-type cells prior to heat shock. Normalized expression levels (log2-scale) of Hsf1 targets were compared between JDP mutants and wild-type (wt) cells after 10 and 60 min post heat shock (C). Alternatively, induction factors of mRNA expression (ratio mRNA levels at 10 min vs 0 min post heat shock) and attenuation factors (ratio mRNA levels at 10 min vs 60 min post heat shock) were compared for each strain separately (D). Figure 3 – Figure Supplement 1 Apj1 promotes the displacement of DNA-bound Hsf1 from HSE. (A) Levels and phosphorylation status of Hsf1 is unaltered in apj1Δ cells. S. cerevisiae wt and apj1Δ cells expressing Hsf1-FLAG3-V5 under control of its native promoter were grown at 30°C till logarithmic growth phase and shifted to 38°C. Total cell extracts were prepared at the indicated timepoints after heat shock and levels of Hsf1 were determined by western blot analysis. Zwf1 levels were determined as loading control. Phosphorylated Hsf1 is upshifted and indicated by “*”. (B) Hsf1 and Apj1 occupancies at UAS regions of Hsf1-dependent heat shock gene loci. Occupancies were determined at the indicated time points after heat shock (30°C to 39°C) by a ChIP as [file media-1.pdf]

Figure 1 - Figure Supplement 1

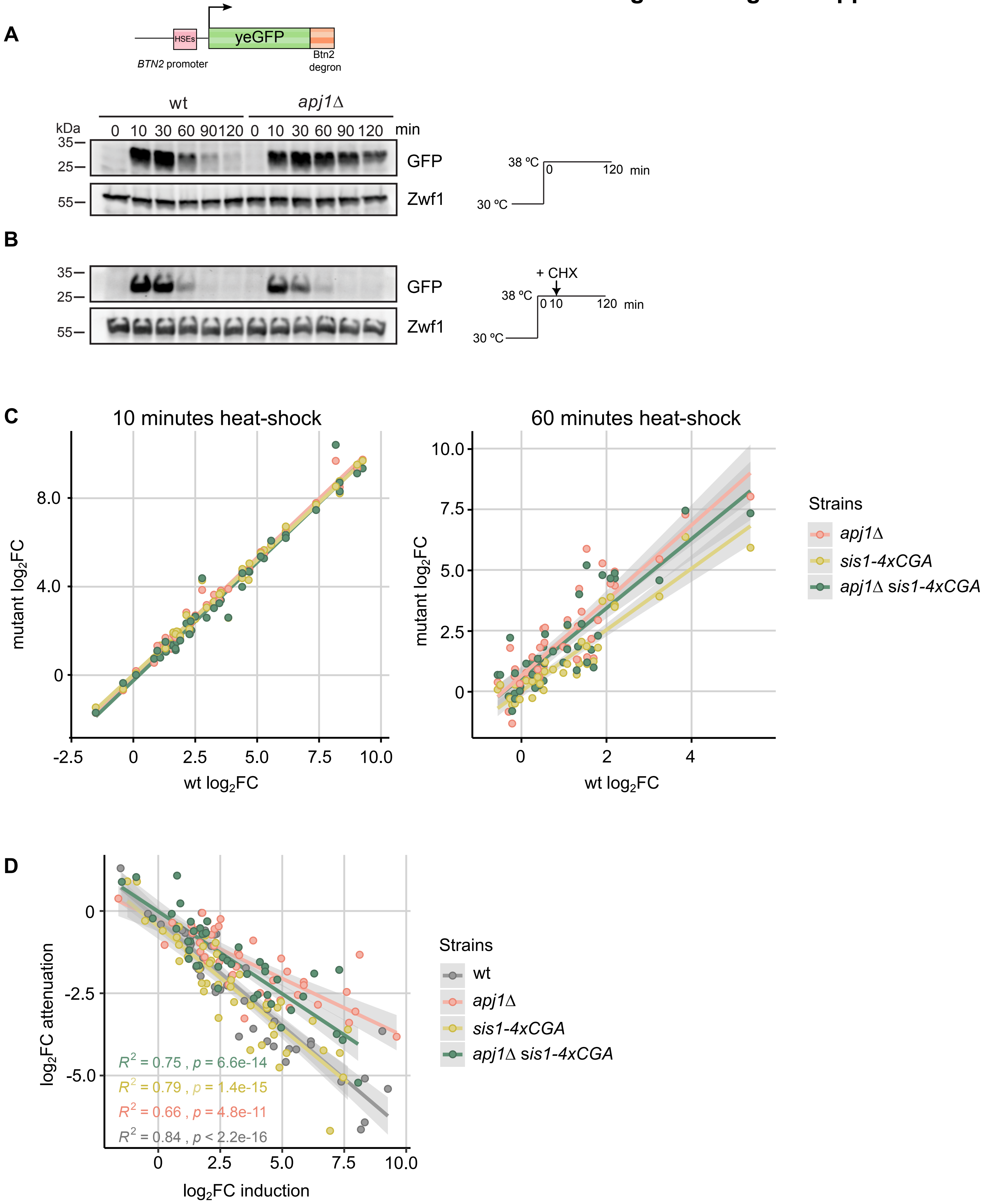

Figure 3 - Figure Supplement 1

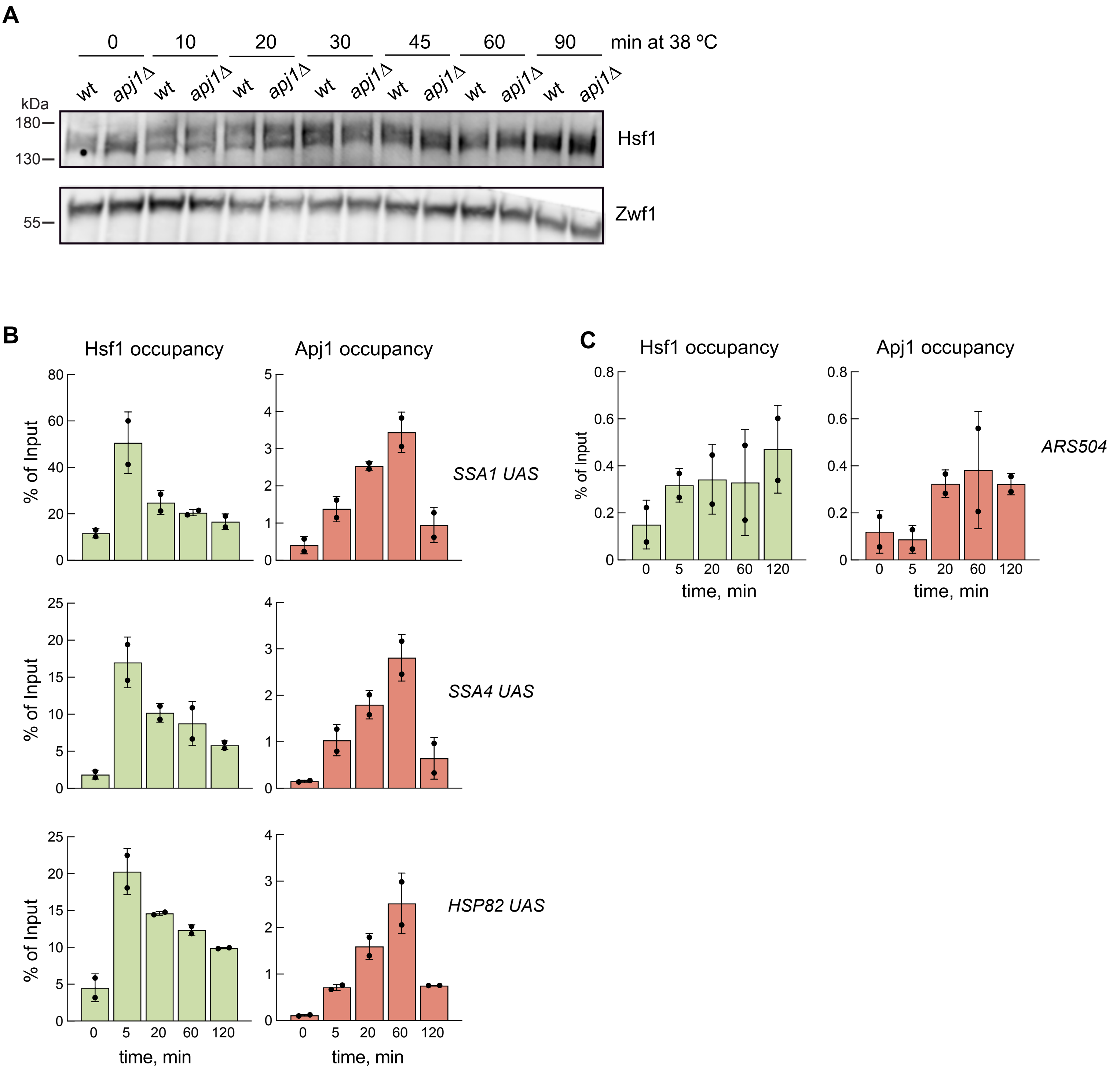

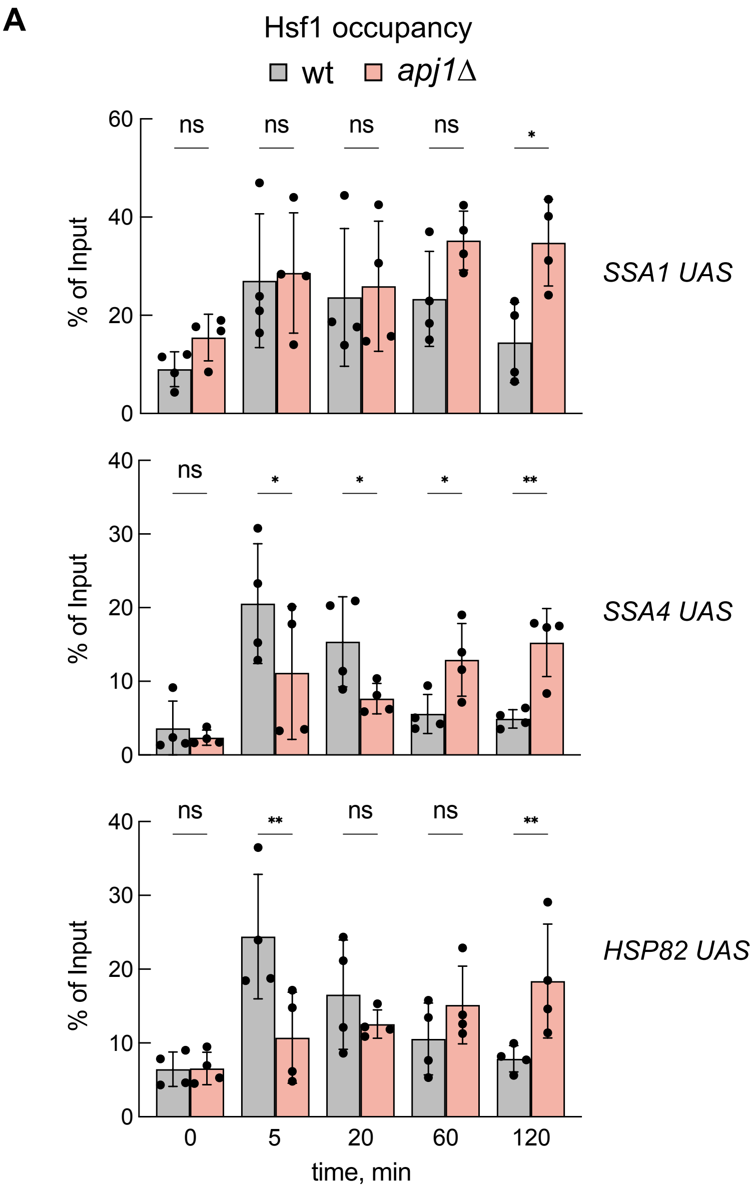

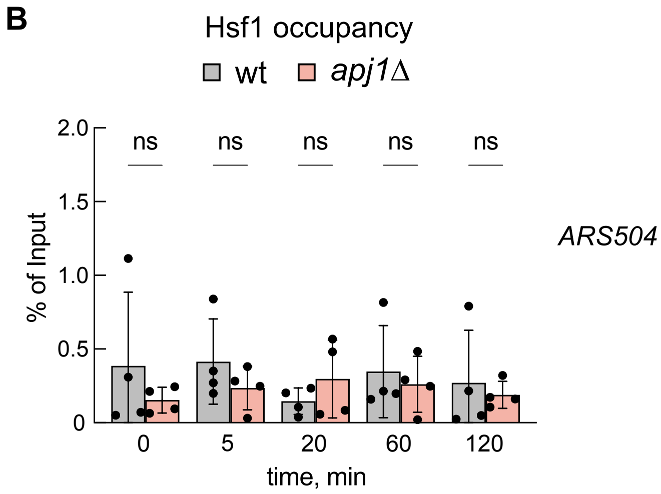

Figure 4 - Figure Supplement 1

Figure 4 - Figure Supplement 2

A

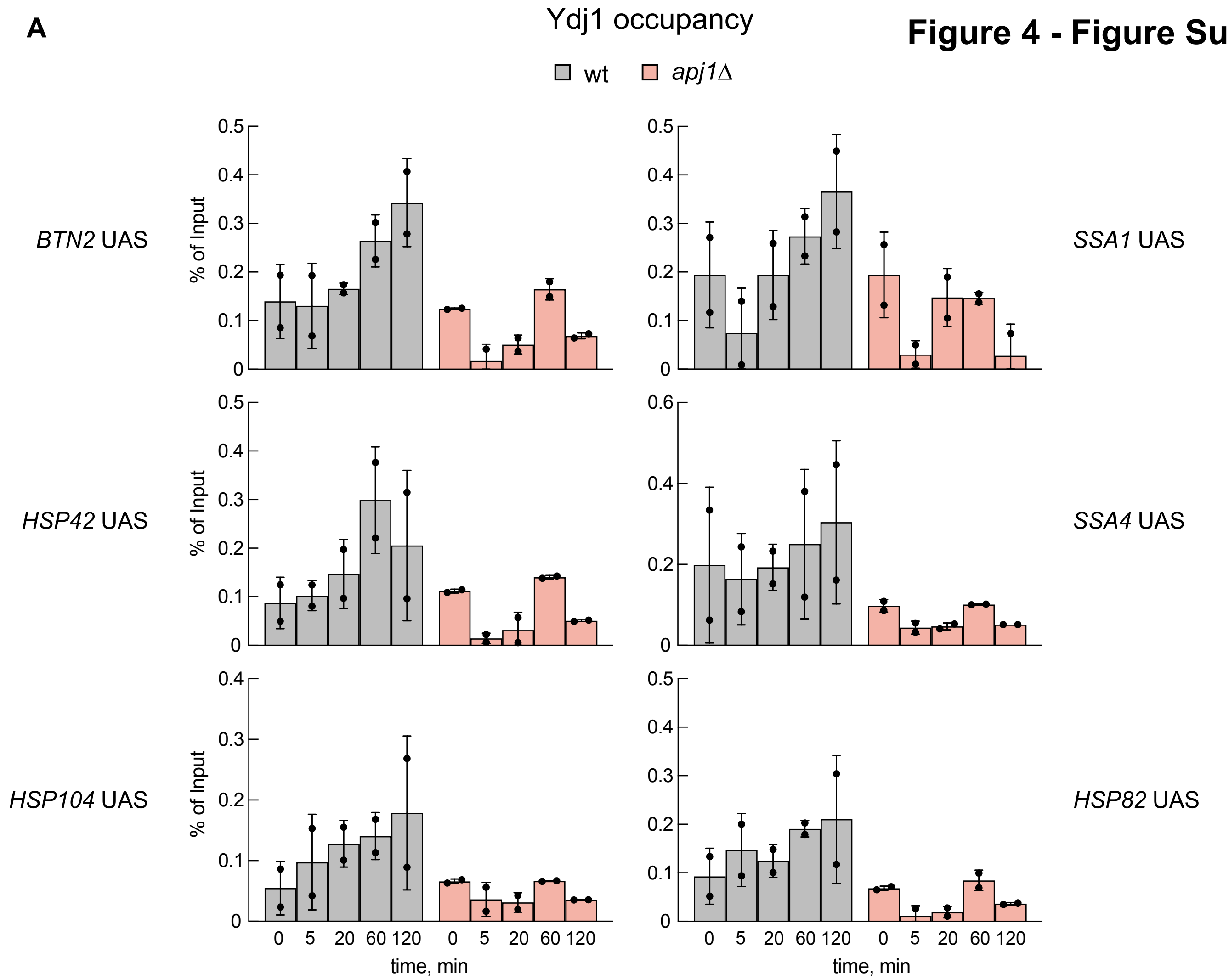

B

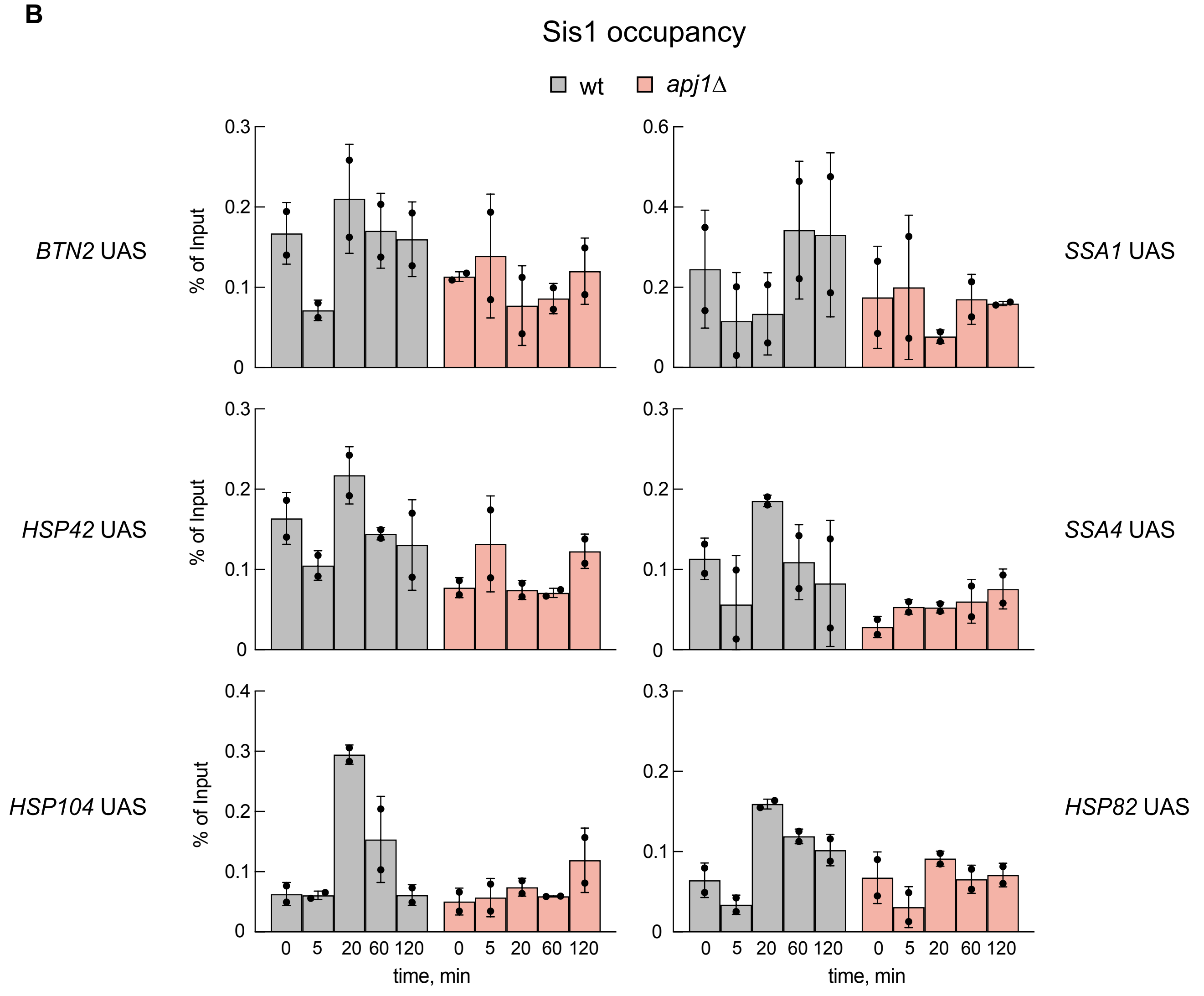

Figure 5 - Figure Supplement 1

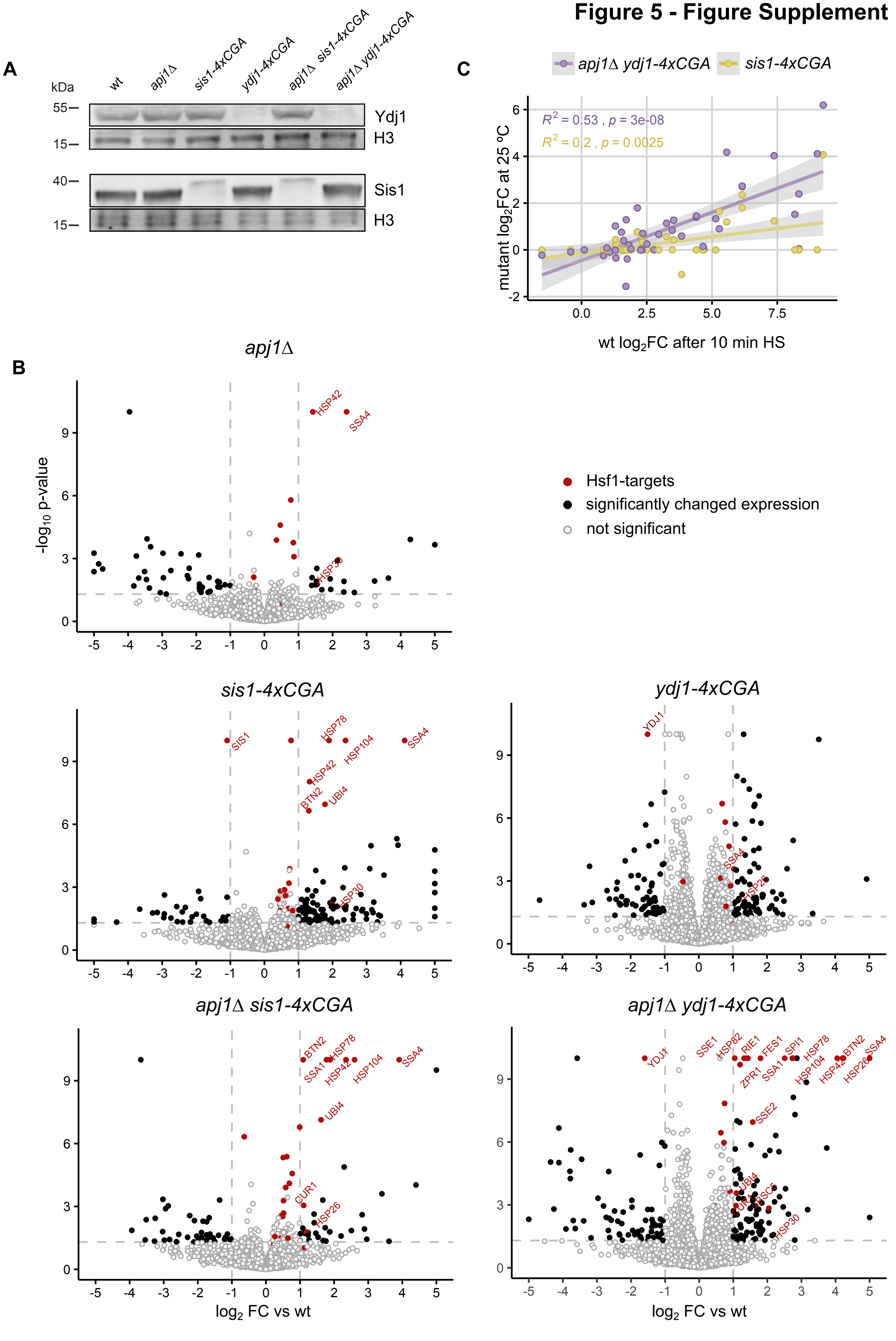

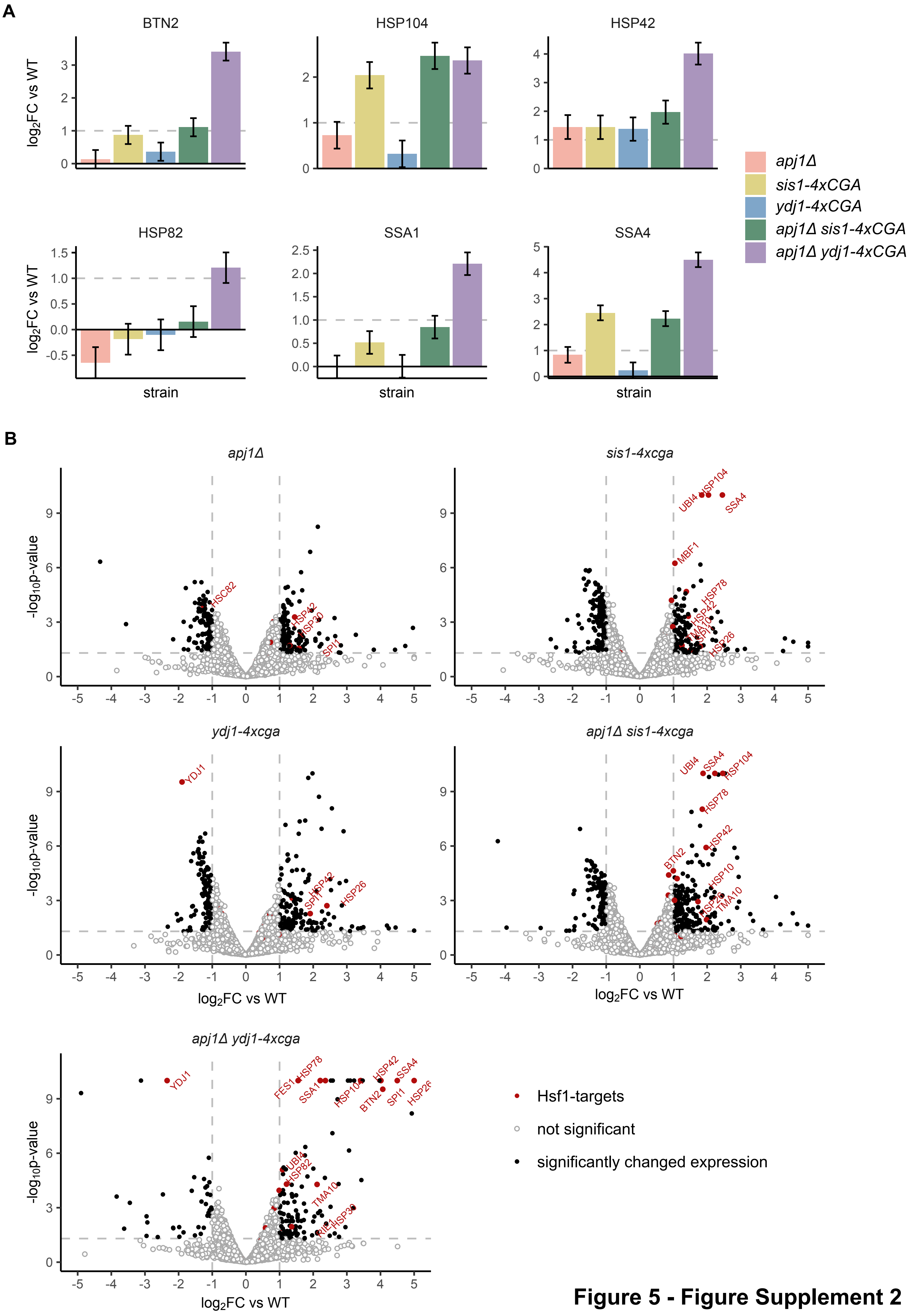

Figure 5 - Figure Supplement 2

Figure 6 - Figure Supplement 1

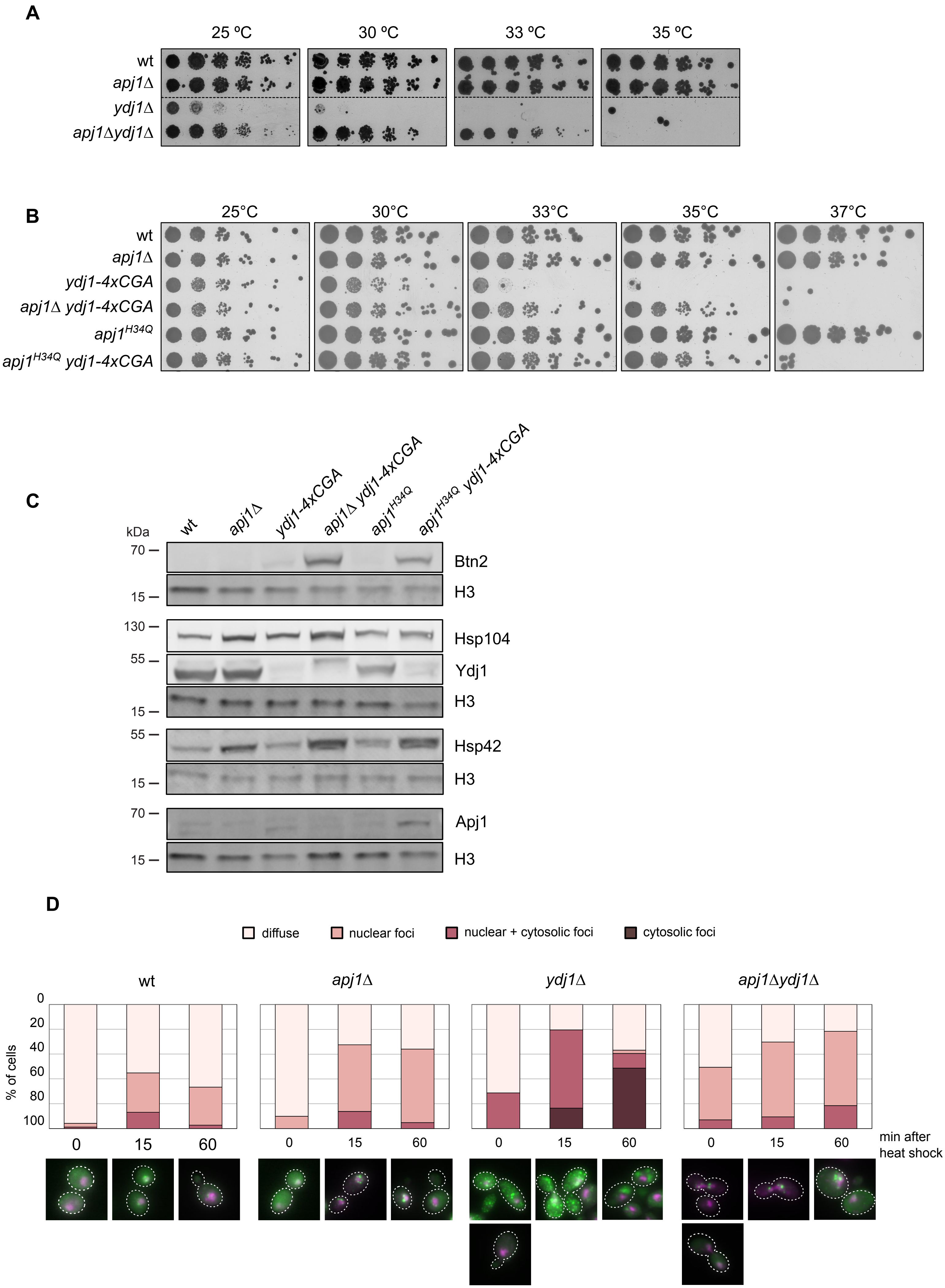

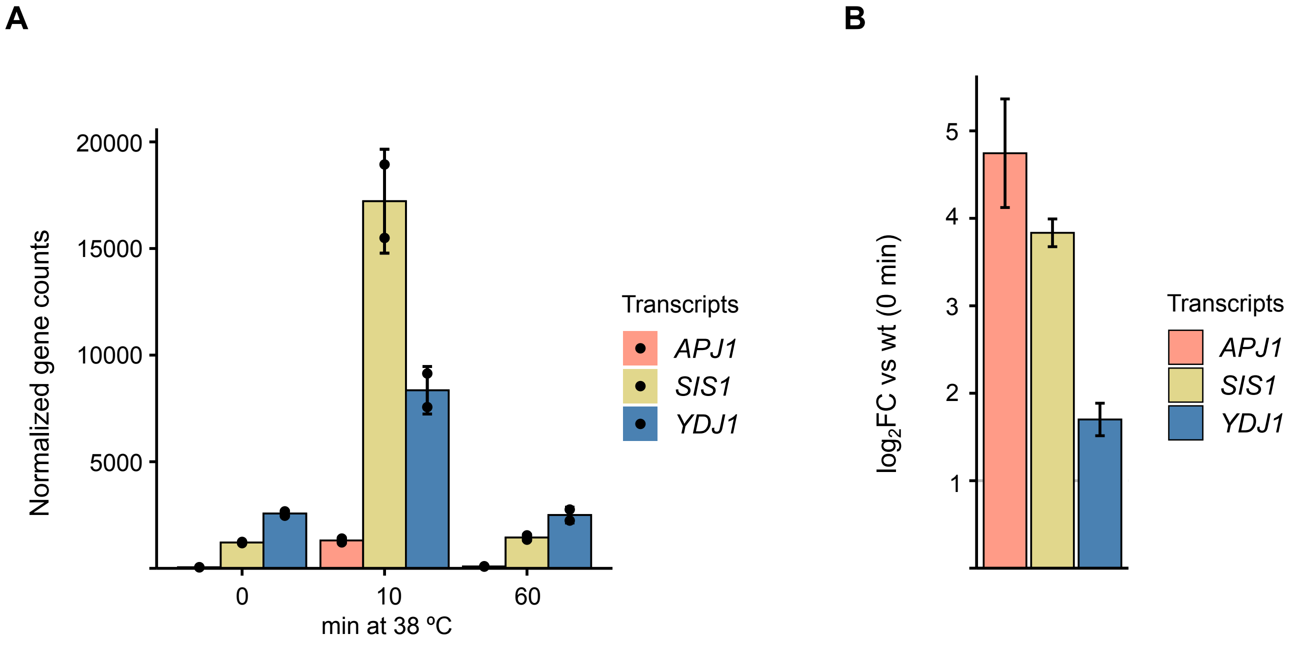

Figure 1 - Figure Supplement 2
