# Supplementary material for: Nuclear and cytosolic J-domain proteins provide synergistic control of Hsf1 at distinct phases of the heat shock response
Source: bioRxiv. 2025 Jul 8:2025.04.14.648540. Preprint. [Version 2] doi: 10.1101/2025.04.14.648540 (PMC12265559; doi:10.1101/2025.04.14.648540)
Supplement: Supplement 2 [file media-2.pdf]

Name	Fenotype	Genotype	Source
BY4741	wt	*MATa his3Δ1 leu2Δ0 met15Δ0 ura3Δ0*	EUROSCARF
W303 - 1B	wt	*MATα leu2-3,112 trp1-1 can1-100 ura3-1 ade2-1 his3-11,15*	EUROSCARF
ASMY09	wt	BY4741 *pdr5Δ::natNT2*	this study
ASMY118	wt	BY4741 *pdr5Δ::natNT2*, *BTN2*prom-yeGFP_Btn2NTD*::hphNT1::HIS3*	this study
ASMY121	*apj1Δ*	BY4741 *pdr5Δ::natNT2*, *BTN2*prom-yeGFP_Btn2NTD*::hphNT1::HIS3*, *apj1Δ::KanMX4*	this study
ASMY144	Hsf1-3xFLAG-V5	BY4741 *pdr5Δ::natNT*2, *hsf1Δ::KanMX4, Hsf1pr-HSF1-3xFLAG-V5::HIS3 (C. glabarata)*	this study
ASMY156	Hsf1-3xFLAG-V5, *apj1Δ*	BY4741 *pdr5Δ::natNT*2, *hsf1Δ::KanMX4, Hsf1pr-HSF1-3xFLAG-V5::HIS3 (C. glabarata)*, *apj1Δ::hphNT1*	this study
FA2216	*hsf1-848*	BY4741 *hsf1-848::KanMX6, cir°*	this study
FA2220	*hsf1-848, apj1Δ*	BY4741 *hsf1-848::KanMX6, apj1Δ::hphNT1, cir°*	this study
CRY063	*apj1Δ*	BY4741, *apj1Δ::HIS3MX6*	this study
CRY066	Hsf1-GFP	BY4741 Hsf1-yeGFP::*HIS3MX6*	this study
CRY068	Hsf1-GFP *apj1Δ*	BY4741 Hsf1-yeGFP::*HIS3MX6, apj1Δ::hphNT1*	this study
CRY108	Hsf1-GFP *ydj1Δ*	BY4741 Hsf1-yeGFP::*HIS3MX6, ydj1Δ::URA3*	this study
CRY110	Hsf1-GFP *apj1Δydj1Δ*	BY4741 Hsf1-yeGFP::*HIS3MX6, apj1Δ::hphNT1, ydj1Δ::URA3*	this study
CRY104	*ydj1Δ*	BY4741 *pdr5Δ::natNT2, BTN2*prom-yeGFP_Btn2NTD::*hphNT1::HIS3,ydj1Δ::URA3*	this study
CRY106	*apj1Δydj1Δ*	BY4741 *pdr5Δ::natNT2, BTN2*prom-yeGFP_Btn2NTD::*hphNT1::HIS3, apj1Δ*::*KanMX4*, *ydj1Δ::URA3*	this study
ASK804	BUD3-HS	BY4741 *UASHS-BUD3*	Chowdhary, S. Kainth, A. et al 2019
GMY008	Apj1-13xMyc	BY4741 Apj1-13XMyc::*KanMX*	this study
GMY011	Apj1-13xMyc, BUD3-HS	BY4741 UASHS-BUD3, Apj1-13xMyc::*KanMX*	this study
GMY019	*apj1Δ*	BY4741 *apj1Δ::hphMX*	this study
YBK26	*apj1Δ*	BY4741, *apj1Δ::hphMX, pdr5Δ::natMX*	this study
GMY062	Sis1-13xMyc	BY4741 Sis1-13XMyc::*KanMX*	this study
GMY063	Sis1-13xMyc, *apj1Δ*	BY4741 Sis1-13XMyc::*KanMX*, *apj1Δ::hphMX*	this study
GMY064	Ydj1-13xMyc	BY4741 Ydj1-13XMyc::*KanMX*	this study
GMY065	Ydj1-13xMyc, *apj1Δ*	BY4741 Ydj1-13XMyc::*KanMX*, *apj1Δ::hphMX*	this study
SMY343	Sis1-GFP	BY4741 pdr5Δ::natMX4, Sis1-yeGFP::*HIS3MX6*	Bukau's lab collection
CRY032	Sis1-GFP, *apj1Δ*	BY4741 pdr5Δ::natMX4, Sis1-yeGFP::*HIS3MX6, apj1Δ::hphNT1*	this study

CRY170	Sis1-GFP, *ypj1Δ*	BY4741 pdr5Δ::natMX4, Sis1-yeGFP::*HIS3MX6, ydj1Δ::URA3*	this study
CRY172	Sis1-GFP, *apj1Δydj1Δ*	BY4741 pdr5Δ::natMX4, Sis1-yeGFP::*HIS3MX6, apj1Δ::hphNT1, ydj1Δ::URA3*	this study
JTY001	wt	W303, GFP-lacI::*HIS3, HSP12-lacO128::URA3, HSP104-lacO256::TRP1, SEC63-13xMyc::KanMX,* POM34-mCherry::*natMX*	Chowdhary, S. Kainth, A. et al 2019
CRY088	*apj1Δ*	W303, GFP-lacI::*HIS3, HSP12-lacO128::URA3, HSP104-lacO256::TRP1, SEC63-13xMyc::KanMX,* POM34-mCherry::*natMX*, *apj1Δ::hphNT1*	this study
CRY164	*ydj1Δ*	W303, GFP-lacI::*HIS3, HSP12-lacO128::URA3, HSP104-lacO256::TRP1, SEC63-13xMyc::KanMX,* POM34-mCherry::*natMX*, *ydj1Δ::BleMX6*	this study
CRY166	*apj1Δydj1*	W303, GFP-lacI::*HIS3, HSP12-lacO128::URA3, HSP104-lacO256::TRP1, SEC63-13xMyc::KanMX,* POM34-mCherry::*natMX*, *apj1Δ*::hphNT1, *ydj1Δ::BleMX6*	this study
LSY009	*ydj1-4xcga*	BY4741, ydj1-4xcga::NatMX	this study
LSY012	*sis1-4xcga*	BY4741, sis1-4xcga::NatMX	this study
LSY020	*apj1Δ ydj1-4xcga*	BY4741, apj1Δ::HIS3MX6, ydj1-4xcga::NatNT2	this study
LSY019	*apj1Δ sis1-4xcga*	BY4741, apj1Δ::HIS3MX6, sis1-4xcga::NatNT1	this study
LSY072	*hsf1-848 ydj1-4xcga*	BY4741 hsf1-848::KanMX6, cir°, ydj1-4xcga::NatNT2	this study
LSY073	*hsf1-848 apj1Δ ydj1-4xcga*	BY4741 hsf1-848::KanMX6, apj1Δ::hphNT1, cir°, ydj1-4xcga::NatNT2	this study
LSY49	*apj1H34Q*	BY4741, *apj1H34Q::HIS3MX6*	this study
LSY067	*apj1H34Q ydj1-4xcga*	BY4741, apj1H34Q::HIS3MX6, ydj1-4xcga::NatNT2	this study
LSY074	wt pRS315 EV	BY4741, pRS315	this study
LSY075	*apj1Δ* pRS315 EV	BY4741, *apj1Δ::HIS3MX6*, pRS315	this study
LSY076	*ydj1-4xcga* pRS315 EV	BY4741, ydj1-4xcga::NatMX, pRS315	this study
LSY077	*apj1Δ ydj1-4xcga pRS315 EV*	BY4741, apj1Δ::HIS3MX6, ydj1-4xcga::NatNT2, pRS315	this study
LSY078	wt TDH3:Sis1	BY4741 pRS315 TDH3:Sis1	this study
LSY079	*apj1Δ* TDH3:Sis1	BY4741, *apj1Δ::HIS3MX6*, pRS315 TDH3:Sis1	this study
LSY080	*ydj1-4xcga* TDH3:Sis1	BY4741, ydj1-4xcga::NatMX, pRS315 TDH3:Sis1	this study
LSY081	*apj1Δ ydj1-4xcga TDH3:Sis1*	BY4741, apj1Δ::HIS3MX6, ydj1-4xcga::NatNT2, pRS315 TDH3:Sis1	this study

Table S2:Plasmids

**Backbone**	**Insert**	Source
pRS315	empty vector	Bukau's Lab collection
pRS315	TDH3:Sis1	Brandman's lab collection
pFA876	pRS315 pApj1 GFP-Apj1-AAA	this study
pFA711	pCU426 pGAL GFP	this study

Table S3: Antibodies

Table S3: Antibodies

Antibody	Dilution	Source	
α-Btn2 (rabbit)	1:5000	Bukau's Lab collection	
α-Hsp42 (rabbit)	1:5000	Bukau's Lab collection	
α-Zwf1 (rabbit)	1:50000	Bukau's Lab collection	
α-Apj1 (rabbit)	1:2000	den Brave's Lab collection	
α-FLAG (mouse)	1:10000	Sigma-Aldrich	
α-H3 (rabbit)	1:10000	Sigma-Aldrich	
α-Sis1 (rabbit)	1:5000	Bukau's Lab collection	
α-Ydj1 (rabbit)	1:5000	Bukau's Lab collection	
α-Hsp104 (rabbit)	1:20000	Bukau's Lab collection	
α-GFP (rabbit)	1:1000	Bukau's Lab collection	
α-cMyc (mouse)	1:360	Santa Cruz Biotechnology	2.5 µl of Ab per sample (ChIP)
α-Hsf1	1:600	Gross' Lab collection	1.5 µl of Ab per sampl (ChIP)
Alkaline Phosphatase Goat Anti-Rabbit IgG, AP1000	1:2500- 1:10000	Vector Laboratories	
Alkaline Phosphatase Goat Anti-Mouse IgG, AP2000	1:2500- 1:10000	Vector Laboratories	

Table S4: RT-qPCR Primer

Figure 3B

Primer Name	Sequence (5' to 3')
pTOS1_RT_for	ACCGACTAATGCGGTCATGGAAAGC
pTOS1_RT_rev	CTTTTCTCGCAAGAAGACTCCAGAATCA
pSSA4_RT_for	GGATATCTTTTGCCCGGTGAGTTG
pSSA4_RT_rev	TGTCGTCAAACTAAGGAGCTTCCC
pUBI4_RT_for	GGAGCATCACACAGCCGTACATC
pUBI4_RT_rev	AAAAGGAGGAACCGCCCTCAAATG
pSSA3_RT_for	GATGCCTATGGAGGTTATGGGTGC
pSSA3_RT_rev	CCCTTCCATTCGTTTCCAATTGTGC
pHSP42_RT_for	CACGCGCTTAAAAGTTCTGGAAGG
pHSP42_RT_rev	AACTAACTTCACAGAGGCCTCCCC
pBTN2_RT_for	GTGGAGCTCGAGAGTTGTATCCAG
pBTN2_RT_rev	CGCCAAGAACTGAAGGCTTCTATG

Figure 3D/E, Figure 4, Figure 3 - Figure Supplement 1, Figure 4 - Figure Supplements 1/2

ARS504 FP	GTC AGA CCT GTT CCT TTA AGA GG
ARS504 RP	CAT ACC CTC GGG TCA AAC AC
HSP104 UAS-267 FP	CTT AAA CGT TCC ATA AGG GGC
HSP104 UAS-216 RP	TGC AGT TCT TTG AGA TGG GCC
HSP82 UAS -394 FP	CCT CTC TCA ACA CAG TAA TCC ATA AAC
HSP82 UAS -242 RP	CTT CCA CGG CGT TCT AGA AAA AAA AG
SSA4 UAS -374 FP	GCC GCA CAT CCA TTC CGG TAT G
SSA4 UAS -312RP	CGG GCA AAA GAT ATC CGC TTT G
SSA1 UAS -428 FP	CGGTGTGTGGATGATGGTTTCATCAT
SSA1 UAS -178 RP	GTCCTCGAAACGATCAGCTAATCTAAATGG
HSP42 UAS -371 FP	GGATATGACATACTTCAATTCAGC
HSP42 UAS -160 RP	CAAGTCTTATATAACTAACTTCACAGAGG
BTN2 UAS -406 FP	GTCATGTAGCACTATTTCAGCC
BTN2 UAS -220 RP	CATTTGTTTTGCCACTTTACTTCG
